# Time of exposure and assessment influence the mortality induced by insecticides against metabolic resistant mosquitoes

**DOI:** 10.1186/s13071-024-06190-z

**Published:** 2024-03-02

**Authors:** Olukayode G. Odufuwa, John Bradley, Safina Ngonyani, Ahmadi Bakari Mpelepele, Isaya Matanila, Joseph B. Muganga, Rune Bosselmann, Ole Skovmand, Zawadi Mageni Mboma, Sarah Jane Moore

**Affiliations:** 1 Vector Control Product Testing Unit (VCPTU) Ifakara Health Institute, Environmental Health, and Ecological Sciences, P.O. Box 74, Bagamoyo, Tanzania; 2 Vector Biology Unit, Department of Epidemiology and Public Health, Swiss Tropical & Public Health Institute, Kreuzstrasse 2, Allschwill, 4123 Basel, Switzerland; 3 Faculty of Science, University of Basel, Petersplatz 1, 4001 Basel, Switzerland; 4 MRC International Statistics and Epidemiology Group, London School of Hygiene and Tropical Medicine (LSHTM), London, WC1E 7HT UK; 5 Vegro Aps, Copenhagen, Denmark; 6 MCC47, Montpellier, France; 7 The Nelson Mandela African Institution of Science and Technology (NM-AIST), Tengeru, P.O. Box 447, Arusha, Tanzania

**Keywords:** Insecticide, Deltamethrin, Pyrethroid, Piperonyl butoxide (PBO), Cone bioassay, Time, ITNs, Malaria, Tanzania

## Abstract

**Background:**

Increasing metabolic resistance in malaria vector mosquitoes resulted in the development of insecticide-treated nets (ITNs) with active ingredients (AI) that target them. Bioassays that accurately measure the mortality induced by these AIs on ITNs are needed. Mosquito metabolic enzyme expression follows a circadian rhythm. Thus, this study assessed (i) influence of the time of day of mosquito exposure and (ii) timing of assessment of mortality post exposure (24 and 72 h) to ITNs against vectors that are susceptible to pyrethroids and those with metabolic and knockdown resistance mechanisms.

**Methods:**

Two cone bioassay experiments were conducted following World Health Organization (WHO) guidelines. Firstly, on ITNs incorporated with 2 g AI/kg of deltamethrin (DM) alone, or combined with 8 g AI/kg piperonyl butoxide (PBO) synergist, during the day (9:00–14:00 h) and repeated in the evening (18:00–20:00 h). This was followed by a confirmatory experiment during the afternoon (12:00–14:00 h) and repeated in the night (22:00–24:00 h) using mosquitoes unexposed or pre-exposed to PBO for 1 h before exposure to DM ITNs. Each net piece was tested with a minimum of eight cones per time (N = 24). The outcome was mortality after 24 h (M24) or 72 h (M72) of holding.

**Results:**

The cone bioassays performed using metabolic resistant mosquitoes during the evening showed significantly lower M24 than those performed in the day for DM: odds ratio (OR) 0.14 [95% confidence interval (CI) 0.06–0.30, *p* < 0.0001] and DM PBO [OR 0.29 (95% CI 0.18—0.49, *p* < 0.0001). M72 was higher than M24 for metabolic resistant mosquitoes exposed to DM [OR 1.44 (95% CI 1.09–1.88), *p* = 0.009] and DM PBO [OR 1.82 (95% CI 1.42–2.34), *p* < 0.0001]. An influence of hour of experiment and time of assessment was not observed for mosquitoes that had knockdown resistance or that were pyrethroid-susceptible.

**Conclusions:**

Time of day of experiment and hour of assessment of delayed mortality after exposure of mosquitoes are important considerations in evaluating insecticides that interact with mosquito metabolism to counter metabolic resistant mosquitoes. This is important when evaluating field-aged ITNs that may have lower concentrations of AI.

**Graphical Abstract:**

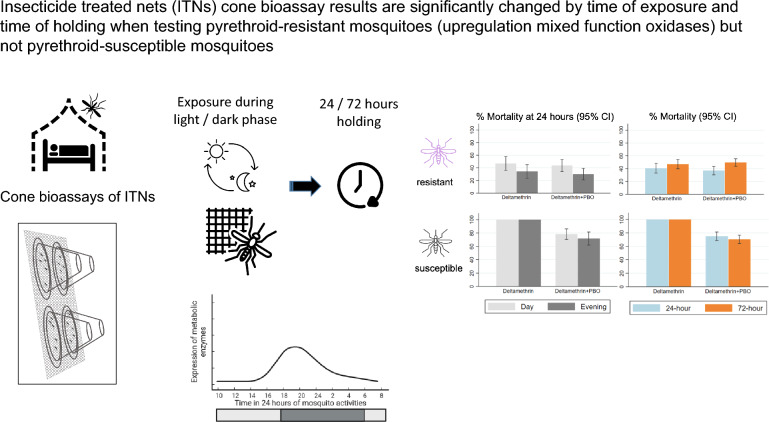

**Supplementary Information:**

The online version contains supplementary material available at 10.1186/s13071-024-06190-z.

## Background

Active ingredients (AI), especially those that belong in the pyrethroid insecticide class, a key public health intervention used in controlling mosquitoes to prevent malaria [1], dengue [2], and nuisance biting [3]. Deltamethrin (DM) is one of the pyrethroid insecticides listed by the World Health Organization (WHO) for public health use [4]. This insecticide is often coated or incorporated into insecticide-treated nets (ITNs) and was sprayed on walls and thatch roof of houses in the form of indoor residual spraying (IRS) [5]. Pyrethroid insecticides work by incapacitating mosquitoes (knockdown), interfering with blood feeding or killing mosquitoes [6], thereby reducing vector-human contact and reducing the size of and average age of the local population of mosquitoes to reduce malaria [7].

The widespread use of the same insecticide class increases selection pressure for resistance [8]. Often, from the larval stage, mosquitoes are exposed to pesticides sprayed in and around their breeding sites for plant protection [9–11]. Larval exposure and increased use of public health pesticides [12] have led to the development of several mechanisms of resistance in mosquitoes [13]. The most wide spread are knockdown resistance (KDR) and metabolic resistance [14]. KDR resistance is caused by mutation of genes in the voltage-gated sodium channel resulting in reduced binding of pyrethroid and Dichlorodiphenyltrichloroethane (DDT) [15]. Metabolic resistance [16] increases the mosquitoes’ ability to detoxify insecticides [8]. It has been shown that there is a circadian component to the upregulation of detoxification enzymes [17]. Resistance means that mosquitoes are able to survive contact with the ITNs and IRS and continue with the transmission of pathogens, contributing to the stalling of progress in the fight against malaria [4]. To combat this public health problem, piperonyl butoxide (PBO), a synergist AI which restores the effect of pyrethroid insecticides by inhibiting metabolic enzymes involved in pyrethroid detoxification, has been shown to reduce malaria prevalence in areas where resistant malaria vectors predominate [18, 19].

New AIs must undergo robust testing for safety and efficacy, following WHO guidelines and procedures, before they are approved for public health use [20, 21]. The first part of the testing takes place in a laboratory. These proof of concept tests feed into how further testing, including regeneration time and wash resistance for ITNs, residual efficacy for IRS and experimental hut trials for both ITNs and IRS, will be conducted. The laboratory tests of neurotoxic insecticides mainly utilise forced contact cone bioassays, aiming to assess the bioefficacy [knockdown after 60 minutes (min) and mortality after 24 hours (h)] of insecticides after 3-min exposure for ITNs and 30-min for IRS. Cone bioassays may also be used to monitor the continued bioefficacy of ITNs after use during operational monitoring [22]. These tests generally require ITNs or IRS to meet set criteria (inducing ≥ 80% mortality after 24 h) to be considered bioefficacious [20]. The new WHO guidelines for laboratory and quality testing of ITNs require proportion of mortality of mosquitoes exposed to the test ITNs to be within 5% of the proportion of mortality in the reference ITNs [23]. To be reproducible [24] and to obtain precise estimates of bioefficacy, it is important to control the many factors that may affect the outcome of these tests, including the time of day when the mosquitoes are exposed to the AI because of circadian rhythm [17, 25] and how long the mosquitoes are monitored after exposure [26]. Therefore, vector control products may be erroneously rejected or listed, simply because of the testing conditions. This study examined (i) the influence the time of day of mosquito exposure and (ii) the time post exposure of measuring mortality (at either 24 or 72 h) from DM, with and without the synergist PBO against mosquitoes of differing resistance status. The study aims to contribute to the standardization of cone bioassay procedures for assessing new AIs that may be metabolically detoxified or interfere with mosquito metabolism to restore the effect of an insecticide.

## Methods

### Description of test facility

Cone bioassays were conducted at the Vector Control Product Testing Unit’s (VCPTU) facility of the Ifakara Health Institute (IHI) in Tanzania, which is Good Laboratory Practice accredited (SANAS GLP0003).

### Study design

**Fig. 1:**
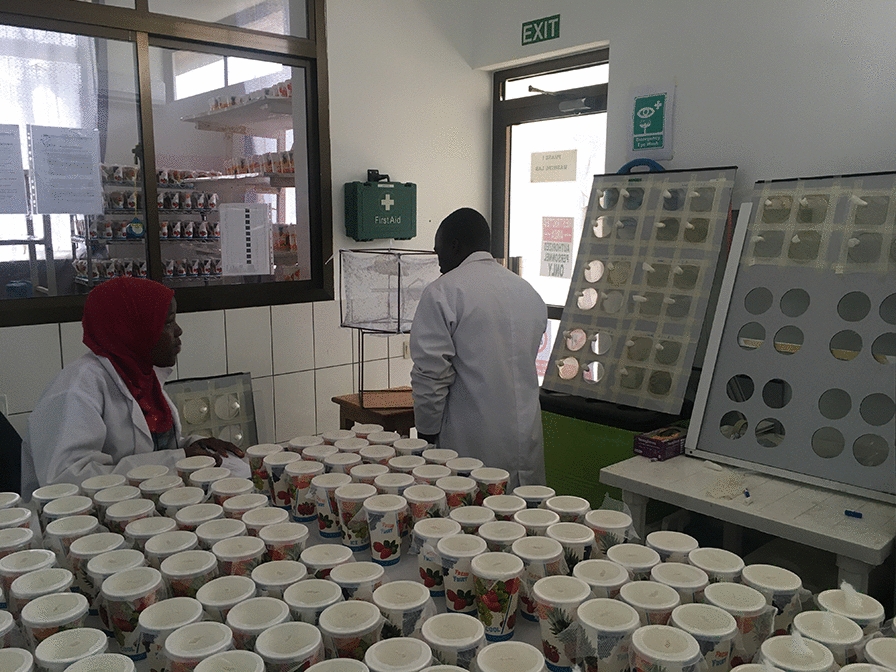
WHO cone bioassay

Net samples (25 cm × 25 cm) were allocated a code by the manufacturer, and both study investigators and technicians were blinded to the allocation until after the data were locked. Cone bioassays (Fig. 1) were conducted following WHO 2013 guidelines for laboratory testing of ITNs [20], with two modifications: the angle of the board was set at 60°, and the samples were placed over a hole cut out from the testing board to maximise mosquito contact with the test net to enable standardized comparison of insecticidal activity [27, 28]. Untreated polyester nets (Safi Net) were used as a negative control to assess experiment quality (control mortality). A minimum of eight replicates (cones) per piece were conducted, although four replicates are the minimum recommended by WHO per ITN sample [20].

### Description of insecticide-treated net

Four treatment arms were tested with three net pieces measuring 25 × 25 cm tested per arm: (i) DM at 2 g AI/kg; (ii) DM at 2 g AI/kg combined with PBO at 8 g/kg, and both incorporated on yarn of 0.152 ﻿millimetres(mm) in diameter nets [for unimproved house modification tool: coverage of the eaves (Insecticide-treated eave nets, ITENs) and window screens, ITWS)] [29], formulated by Intelligent Insect Control, France, and supplied by Vegro Aps, Denmark; (iii) Tsara Soft, a polyester ITN coated with 2 g AI/kg DM and made of 100-denier knitted monofilament High Density Polyethylene (HDPE) fibres, manufactured by NRS Moon Netting FZE; (iv) Tsara Soft, which was tested after mosquitoes had been pre-exposed to WHO insecticide-impregnated papers of 4% PBO concentration. The negative control to monitor the quality of experimental conduct was done using an untreated polyester net, SafiNet, manufactured by A to Z Textile Mills, Tanzania. Net pieces were stored in a temperature-controlled refrigerator at 4 ºC between tests.

### Description of test systems (study mosquitoes)

Each net sample was tested against 2–5-day-old nulliparous sugar-fed mosquitoes. Six strains of mosquitoes were used. Three had metabolic resistance: *Anopheles arabiensis* (Kingani strain), *Culex quinquefasciatus* (Bagamoyo strain) and *An. funestus* (FUMOZ strain). One had KDR resistance: *An. gambiae* (Kisumu strain); two were pyrethroid-susceptible: *An. gambiae* (Ifakara strain) and *Aedes aegypti* (Bagamoyo strain). Strains’ susceptibility level and origin are listed in Additional file 1. The colonies are maintained following relevant Standard Operating procedures (SOPs) adapted from Malaria Resources (MR4) guidance [30]. Mosquitoes used in the study were reared under laboratory conditions of 27 ± 2 °C temperature and 55–100% relative humidity. Mosquito larvae were reared at a density of 200/l and fed with Tetramin fish food. Adults were provided 10% sugar solution ad libitum supplemented with cow blood from a membrane for egg laying.

### Cone bioassay procedure

Two sets of cone bioassay experiments were performed. The first tests were conducted in September and October 2020 using 0.152-mm-diameter nets incorporated with DM only or DM combined with PBO. Tests were conducted during the day (09:00–14:00 h), representing completion time for a cone bioassay experiment and repeated on the same samples in the evening (18:00–20:00 h), given that *Anopheles* mosquitoes have been reported to detoxify insecticides more intensively in the evenings and nights than during the day [17]. Therefore, three strains were used, two metabolic resistant strains, *An. arabiensis* and *Cx. quinquefasciatus*, and one pyrethroid-susceptible strain, *Ae. aegypti* (diurnal activity). The second tests were conducted in February and March 2023 using Tsara Soft ITNs with or without mosquitoes pre-exposed to PBO for 1 h before exposure. This was done to more narrowly identify the hours of the effect observed in the first tests and to extend the tests to more mosquito strains. Tests were conducted during the afternoon (12:00–14:00 h) and repeated on the same samples in the night (22:00–24:00 h), because many vector mosquitoes are active at night [31]. As in the previous experiments, metabolic resistant *An. arabiensis* and *Cx. quiquefasciatus* and susceptible *Ae. aegypti* were tested. In addition, metabolic resistant *An. funestus*, KDR resistant *An. gambiae* and pyrethroid-susceptible *An. gambiae* were tested.

For both tests, mosquitoes were acclimatised for a minimum of 30 min in the test room before they were exposed to test net pieces. Nets were taken from the fridge and attached on a testing board using a masking tape then allowed to return to room temperature for a minimum of 1 h before testing. Following WHO guidelines, four standard WHO cones were placed on each net piece (overlaid on a hole cut into the board to maximise mosquito contact [27]) and attached to the board with a masking tape. Five mosquitoes were aspirated using a syphon into each cone and temporarily sealed using a plastic bung (cotton wool inserted into a tip of a latex glove) for 3 min [20]. After the exposure, the mosquitoes were removed gently from the cones and kept in paper cups (one cup per replicate) provided with cotton wool moistened with 10% sugar solution. Mosquitoes were held under insectary conditions in their respective cups in a holding room to measure delayed mortality at 24 h (M24) and 72 h (M72). A minimum of 120 mosquitoes per species were exposed per time of experiment (that is, day, evening, afternoon and night) against each treatment (i.e. 40 mosquitoes in 8 cones applied to 3 net pieces).

### Data management and analysis

Data were collected using paper forms and were double entered into Microsoft Excel. Data were cleaned and analysed using STATA statistical software, version 16.1 (Stata Corp) [32]. Data were cleaned by checking they had been collected accurately according to study protocol: balance in treatment arms, exposure time of 3-min, number of mosquitoes exposed, number of mosquitoes available after hours of holding to observe delayed mortality, number of replicates and age range of mosquitoes recorded. Where discrepancies were found, raw data sheets were checked and electronic data corrected. Descriptive analysis was conducted on outcomes: M24 and M72. The percentages of the arithmetic mean with respective 95% confidence intervals were estimated. Fixed effect binomial logistic regression was used to estimate the influence of time of mosquitoes’ exposure in the cone bioassay and time of assessment on mortality. For the influence of exposure time analysis, the model was adjusted for mosquito strains as a fixed effect given that strains vary from one another in terms of circadian rhythm [33] and resistance to insecticide (Additional file 1), and the time of experiment was the fixed effect of interest. For the time of mortality assessment analysis, the model was adjusted for both mosquito strains and time of experiment as fixed effects because of variation in the circadian rhythm observed among strains [17], and the holding time (24 vs 72 h) was the fixed effect of interest.

## Results

### Experimental validity

The testing and holding rooms ranged from 26.9 °C [interquartile range (IQR): 26.4–27.3] and 64.7% (IQR: 60.0–67.7) relative humidity (RH) during the experiment. Mortality in the untreated net (negative control) was 0% at 24 h and < 2% at 72 h; therefore, there was no need to adjust for control using Abbott’s formula [20].

### Time of day of bioassay conduct is important for metabolic resistant but not susceptible strains

**Fig. 2:**
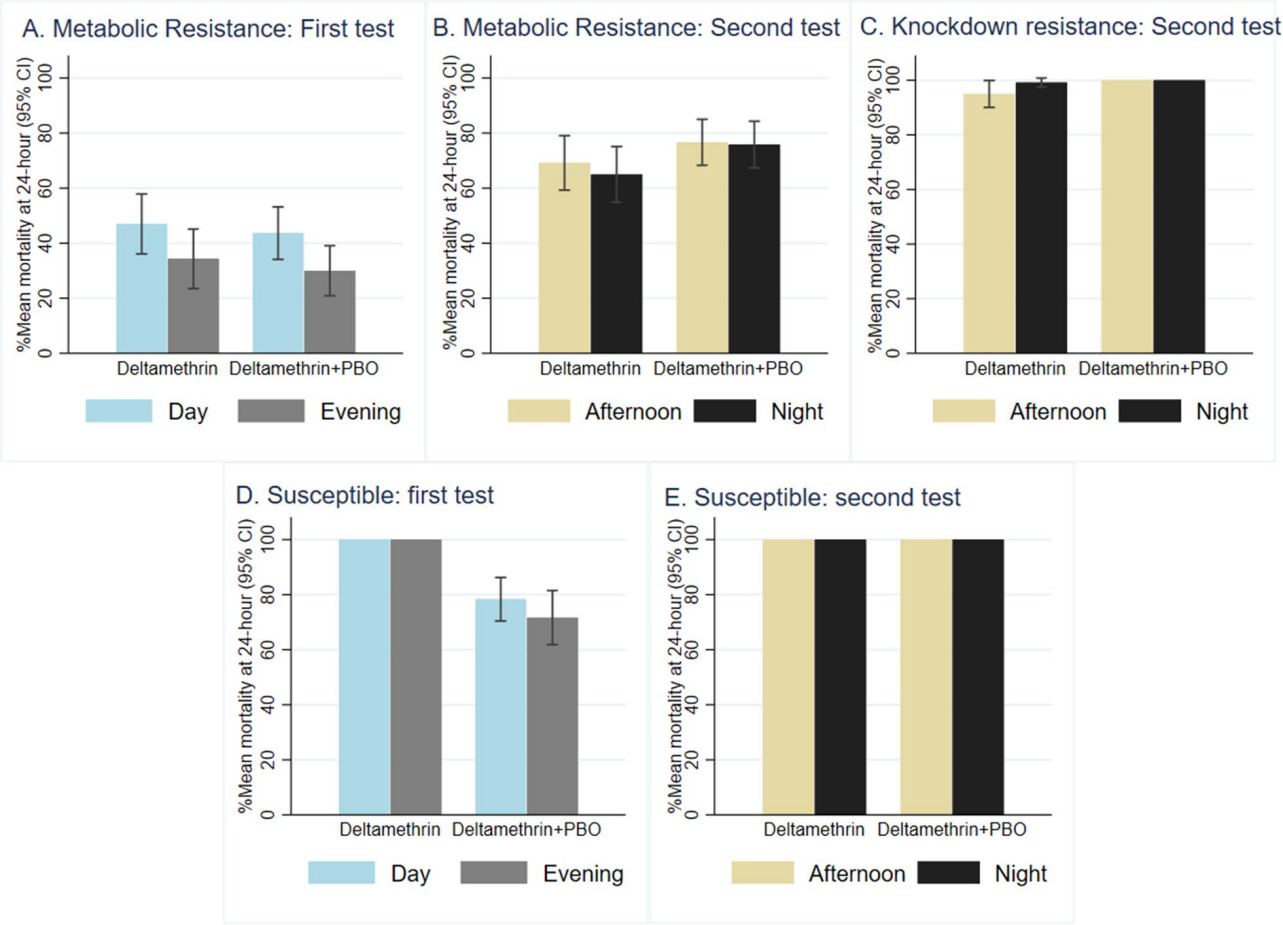
Influence of time of experiment on mortality at 24 h. Percentage arithmetic mean mortality at 24 h with respective 95% confidence interval (CI) for cone bioassays conducted in the first test using incorporated deltamethrin or deltamethrin-PBO nets in day (9:00–14:00 h) vs. evening (18:00–20:00) and in the second test using coated deltamethrin or with pre-exposure to PBO in the afternoon (12:00–14:00 h) vs. night (22:00–24:00 h)

The cone bioassays performed during the evening (18:00–20:00 h) showed significantly lower percentage mortality at 24 h (M24) than those performed in the day (9:00–14:00 h) for metabolic resistant mosquitoes (Table 1). There was no effect of time of exposure observed for mosquitoes with knockdown resistance or those that were fully susceptible to pyrethroid (Fig. 2, Table 1).

**Table 1:** Influence of time of experiment on mortality at 24 h

Experiment period	Resistant profile	Insecticides and synergist	Time of assay	Total	Dead	% Mortality at 24 h (95% CI)	OR^a^ (95% CI)	*P*-value
First	Metabolic resistant (*Anopheles arabiensis* and *Culex quinquefasciatus*)	DM	Day	300	141	47.0 (36.1–57.9)	1.00	
			Evening	300	103	34.3 (23.5–45.1)	0.14 (0.06–0.30)	< 0.0001
		DM + PBO	Day	300	131	43.7 (34.2–53.2)	1.00	
			Evening	300	90	30.0 (20.9–39.1)	0.29 (0.18–0.49)	< 0.0001
	Fully susceptible (*Aedes aegypti*)	DM	Day	120	120	100	1.00	
			Evening	120	120	100	1.00	**–**
		DM + PBO	Day	120	94	78.3 (70.5–86.2)	1.00	
			Evening	120	86	71.7 (61.9–81.4)	0.70 (0.39–1.26)	0.234
Second	Metabolic resistant (*Anopheles arabiensis*, *An. funestus* and *Cx. quinquefasciatus*)	DM	Afternoon	360	249	69.2 (59.3–79.0)	1.00	
			Night	360	234	65.0 (54.9–75.1)	0.40 (0.19–0.82)	0.013
		DM + PBO	Afternoon	360	276	76.7 (68.3–85.0)	1.00	
			Night	360	273	75.8 (67.4–84.3)	0.89 (0.51–1.55)	0.669
	Knock down resistant (*An. gambiae*)	DM	Afternoon	120	114	95.0 (90.1–99.9)	1.00	
			Night	120	119	99.2 (97.5–100)	6.26 (0.74–52.84)	0.092
		DM + PBO	Afternoon	120	120	100	1.00	
			Night	120	120	100	1.00	**–**
	Fully Susceptible (*An. gambiae* and *Aedes aegypti*)	DM	Afternoon	240	240	100	1.00	
			Night	240	240	100	1.00	**–**
		DM + PBO	Afternoon	240	240	100	1.00	
			Night	240	240	100	1.00	**–**

Mortality in the negative control was  0% at 24 h

Results are from a fixed effect binomial logistic regression

Day or afternoon time is set as the reference, and mosquito strains are set as fixed effects

^a^ Adjusted Odds ratio

First experiment tested ITENs and ITWS fabric and second experiment tested ITNs fabric and analysed separately

In the comparison of ITNs tested in the day or evening, for nets treated with DM only, the difference was 13%: 34% (103/300) vs. 47% (141/300), odds ratio (OR) = 0.14 (95% confidence interval: 0.06–0.30, *p*-value < 0.0001). For DM-PBO synergist nets, the difference was 14% [30% (90/300) vs. 44% (131/300), OR = 0.29 (95% CI 0.18–0.49, *p* < 0.0001)]. Similarly, a significantly lower M24 was measured for cone bioassay performed in the night (22:00–24:00 h) than in the afternoon (12:00–14:00 h) for DM only [65% (234/360) vs. 69% (249/360), OR = 0.40, (95% CI 0.19–0.82), *p* = 0.013]. There was no significant difference in M24 in cone bioassays conducted on metabolic resistant mosquitoes in the night compared to afternoon against DM when the mosquitoes were pre-exposed to the PBO synergist.

When the influence of time of day on the observed mortality of each individual strain is considered, the differences were consistent. All metabolic resistant strains showed significantly lower mortality when tests were conducted in the evening or night relative to earlier (day or afternoon). There were differences in the magnitude of effect observed with the largest difference in observed mortality among the *Cx. quinquefasciatus* strain that is the most highly resistant (Additional file 2). Pre-exposure to PBO reduced this difference in observed mortality to some extent, and the coated deltamethrin ITN tested against resistant *An. arabiensis* killed all mosquitoes when tested in the afternoon or the night. This indicates a dose-dependent relationship between observed mortality and bioavailability that is mediated by circadian regulation of metabolic enzymes.

### Longer holding time for delayed mortality is important for resistant but not susceptible strains

**Fig. 3:**
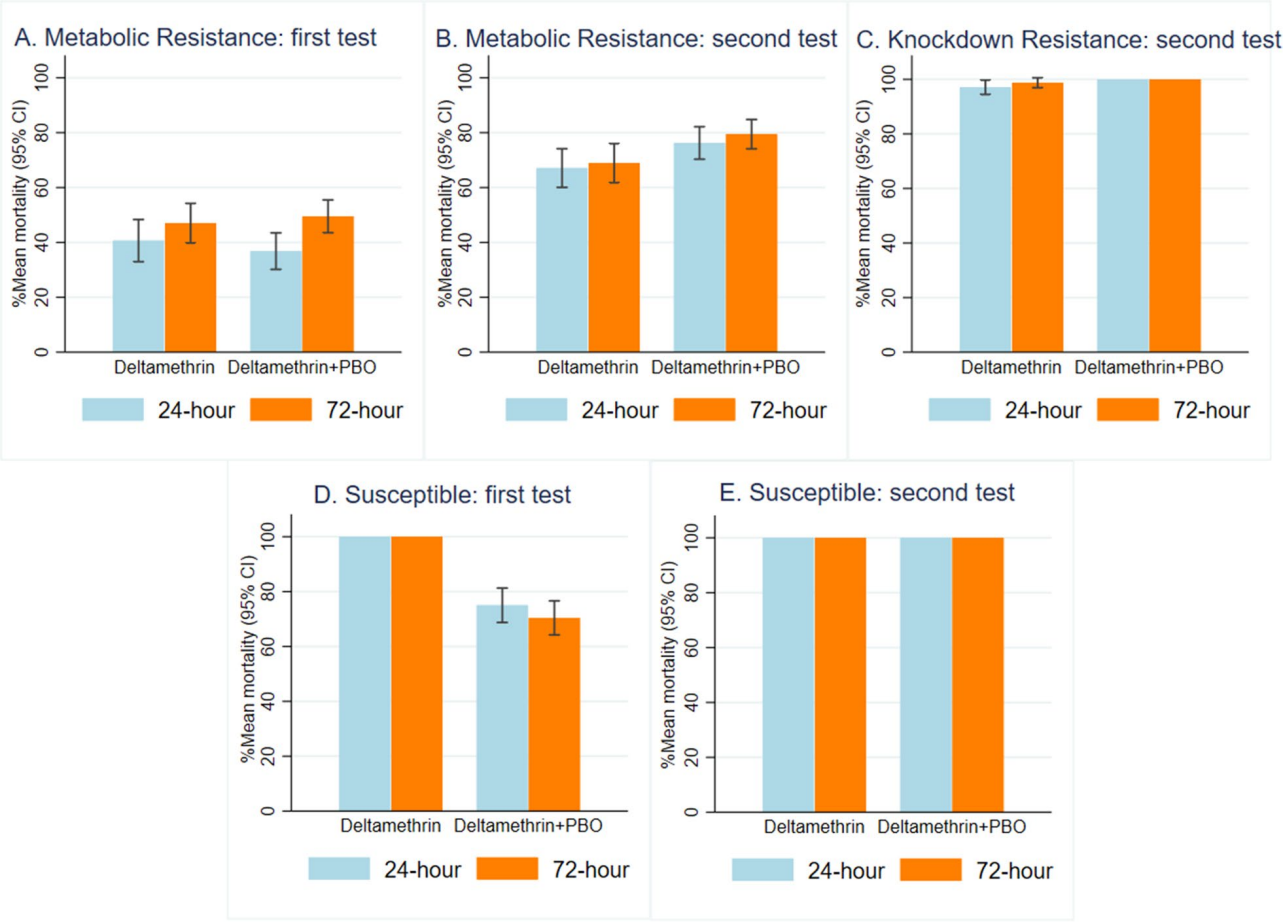
Longer holding time for delayed mortality. Percentage arithmetic mean mortality assessed at 24 h and 72 h with respective 95% confidence intervals. The first test was conducted using incorporated deltamethrin or deltamethrin-PBO net. The second test was conducted using a coated deltamethrin net with or without pre-exposure to PBO

A difference between the timing of assessment on mortality was seen among metabolic resistant mosquitoes only and only in the first tests (Fig. 3), with higher proportion of mortality at 72 h than at 24 h for DM [47% (282/600) vs. 41% (244/600), OR = 1.44 (95% CI 1.09–1.88), *p* = 0.009] and DM combined with PBO synergist [50% (297/600) vs. 37% (221/600), OR = 1.82 (95% CI 1.42–2.34), *p* < 0.0001]. In the second experiment with Tsara Soft or Tsara Soft with pre-exposure to PBO showed very little difference in observed mortality between holding times. Mortality was similar between 24 and 72 h assessment for KDR and susceptible mosquitoes in the first and second experiment (Fig. 3, Table 2).

**Table 2:** Influence of longer holding time on mortality of laboratory-reared mosquitoes

Experiment period	Resistant profile	Insecticides and synergist	Hour of assessment	Total	Dead	% Mortality (95% CI)	OR^a^ (95% CI)	*P*-value
First	Metabolic resistant (*Anopheles arabiensis* and *Culex quinquefasciatus*)	DM	24	600	244	40.7 (33.0–48.4)	1.00	
			72	600	282	47.0 (39.8–54.2)	1.44 (1.09–1.88)	0.009
		DM + PBO	24	600	221	36.8 (30.2–43.5)	1.00	
			72	600	297	49.5 (43.5–55.5)	1.82 (1.42 –2.34)	< 0.0001
	Fully susceptible (*Aedes aegypti*)	DM	24	240	240	100	1.00	
			72	240	240	100	1.00	
		DM + PBO	24	240	180	75.0 (68.8–81.2)	1.00	
			72	240	169	70.4 (64.2–76.6)	0.87 (0.64–1.19)	0.381
Second	Metabolic resistant (*Anopheles arabiensis*, *An. funestus* and *Cx. quinquefasciatus*)	DM	24	720	483	67.1 (60.1–74.1)	1.00	
			72	720	496	68.9 (61.8–76.0)	1.10 (0.87–1.39)	0.410
		DM + PBO	24	720	549	76.3 (70.3–82.2)	1.00	
			72	720	572	79.4 (74.1–84.8)	1.14 (0.93–1.41)	0.205
	Knock Down resistant (*Anopheles gambiae*)	DM	24	240	233	97.1 (94.5–99.7)	1.00	
			72	240	237	98.8 (96.9–100)	1.07 (0.75–1. 52)	0.720
		DM + PBO	24	240	240	100	1.00	
			72	240	240	100	1.00	
	Fully susceptible (*An. gambiae* and *Ae. aegypti*)	DM	24	480	480	100	1.00	**–**
			72	480	480	100	1.01 (0.78–1.30)	0.948
		DM + PBO	24	480	480	100	1.00	**–**
			72	480	480	100	1.02 (0.79–1.31)	0.897

Overall mortality in the negative control was 0% and 1% at 72 h for *Anopheles* *funestus* only

Results are from a fixed effect binary logistic regression; 24-h mortality is set as the reference

Mosquito strains and time of experiment are set as a fixed effect

^a^ Adjusted Odds ratio

First experiment tested ITENs and ITWS fabric and second experiment tested ITNs fabric, and analysis was done separately.

## Discussion

Circadian rhythm governs activity in mosquitoes including mating and locating resources such as sugar, hosts and oviposition [34]. All of these behaviours require flight that is energetically demanding, and many genes are upregulated in the evening and night when mosquitoes are more active [17], resulting in upregulation of one or more detoxification enzymes that affects mosquito resistance to pyrethroids [35] and DDT [17]. This is the likely reason for the observed lower mortality of mosquitoes that were exposed to insecticides during the evening (30%) than during day (44%) among the mosquito strains known to have metabolic resistance to pyrethroids. The metabolic resistant mosquitoes used in this study are nocturnal; therefore, they are more active from the evening, and studies have shown the linkage of upregulating metabolic detoxification enzymes to clock genes [17, 25]. As PBO restored susceptibility of *An. arabiensis* colony in the susceptibility testing, but only partially restored susceptibility of *Cx. quinquefasciatus*, it is possible that an additional underlying mechanism like gluthathion transferase, which is not blocked by PBO, may also exist in the *Cx. quinquefasciatus* strains tested in addition to elevated oxidases (Additional file 2).

The effect of time of exposure of mosquitoes in cone bioassay on mortality was not seen for KDR mosquitoes because of the different mode of action of the resistance mechanism. The developed target site insensitivity is caused by a point mutation of the genes and confers resistance to DDT and pyrethroid; it is not related to metabolic regulation [15]. Studies have reported no correlation between the presence of the KDR genes and mortality to type II pyrethroids on the individual level [36, 37]. In the study, *An. gambiae* s.s. (Kisumu, KDR) mosquitoes were sensitive to the AI tested (deltamethrin) in the phenotype resistance profile confirmation done at the time of bioassays (Additional file 1). This is reflected in the observation that > 95% of the mosquitoes died at both the night and afternoon exposure time.

There was no significant effect of time on susceptible mosquitoes, although the estimate of the effect was in the direction of lower mortality for the evening experiment for *Ae. aegypti* mosquitoes in the first experiment. This species is more active during the day and has been shown to have a circadian rhythm correlation with resistance to pyrethroid [35]. The observation in our study is as a result of chance as the mosquitoes used were not metabolic resistant to insecticide. It is possible that there could have been evidence of an effect if the sample was larger and compared to other diurnal species that were metabolic resistant. In the second experiment, it was not possible to discern whether or not circadian regulation of detoxification enzymes contributed to the observed differences in the time of testing due to the higher dose of insecticide used on the coated nets. It is possible that at low concentrations of pyrethroids a similar difference might be observed between assays conducted during the day and those conducted during the active phase of the insect’s circadian rhythm. Therefore, confirmatory dose response studies should be done in the day and evening even with susceptible strains to measure whether the same phenomenon would be observed for susceptible insects at low concentrations of pyrethroids.

The full expression of insecticidal activity takes > 24 h [38]. This was more pronounced in the first test (Additional file 3) because of the nature of the nets used, with incorporation technique and higher mesh size [39]. The combination of all these factors might have resulted in efficacy of the nets tested in the first test similar to “sub-lethal dose”. Sub-lethal dose of insecticides has been shown to impact the sensory and neural senses of mosquitoes, such that upon exposure, disorientation, reduced feeding and overexpression of enzymes that negatively affect the fitness and life span of mosquitoes have been observed. Mortality continues to increase until it reaches 100% overtime [40]. When exploring pyrethroid insecticides and new AI that may require a longer time to express its full insecticidal effect, e.g. chlorfenapyr [41], it is important that the mortality induced by the pyrethroid only comparator be explored at the same time to be able to see the additional benefit of the second AI. However, the experiment was not conducted to measure the optimal time for the maximal effect of pyrethroid mediated by PBO, but the estimated ORs were higher for PBO products than DM measured at 72 h, indicating that this should be further explored. There is a concern that transmission may still be ongoing from infected mosquitoes after exposure to insecticide within 72 h after exposure, so faster acting AIs are deemed preferable to slower acting ones. However, slow-acting insecticides are still likely to control malaria through reduced mosquito population size and age [42, 43], as has been observed in recent trials of chlorfenapyr ITNs [44, 45]. A possible investigation would be to explore how many times uninfected and insecticide-exposed mosquitoes seek blood meals after 24 h up to 72 h and more after sub-lethal exposure to insecticide [46].

In the first experiment, the mortality observed for DM was 4% higher than that of DM PBO ITNs against metabolic resistant mosquitoes as well as 25% higher against susceptible *Ae. aedes* mosquitoes. These results do not agree with the resistance testing (Additional file 1) with PBO restoring the efficacy of pyrethroids. However, the classic test of a synergist effect with PBO prescribes that the PBO is added at relatively high concentration, e.g. 4% PBO to 0.005% DM, and 1 h before the pyrethroid [47]. This is not possible for ITNs and IRS, where they will always be presented simultaneously and often at lower ratios of PBO to pyrethroid in the range of 7 to 0.5 PBO to 1 pyrethroid. The predictive value of PBO for overcoming resistance based on pre-exposure to high concentration of PBO is not designed to predict the impact of a vector control tool but to ascertain the likelihood of resistance existing in a mosquito population [47]. Nikpour et al. found that a ratio < 3:1 PBO to DM did not increase mortality or durability of IRS sprays [48]. The nets used in the first experiment had a ratio of 4:1 PBO to DM with a target surface concentration of 8:1; therefore, the release rate of PBO was double that of DM. Over time, it is possible that the PBO was lost before the DM. Chemical confirmation of results was not performed, which is a limitation of the study.

About 69% (396/576) of the observations recorded during the second experiments were below the WHO’s recommended range of 80 ± 20% RH [23]. The majority of these observations [73% (288/396)] were recorded during the afternoon experiment when the temperature was high because of the sunshine. An air conditioner was used to regulate the temperature; however, this decreased the RH in the test room despite the presence of a humidifier and bowls filled with water. It is possible that this might have contributed to the mortality observed because of the negative correlation found between mortality and humidity in this study (Pearson correlation = − 0.0753, *p* = 0.0230) It is known that low humidity can affect mosquito survival [49]. However, the impact was likely small  because the mortality recorded in negative control was < 2% at 72 h [23].

It is clear that conducting cone bioassays on metabolic resistant mosquitoes in the early evening ensures accurate measurement of the mortality of metabolic resistant mosquitoes exposed to pyrethroid and PBO synergist. Therefore, standardization of cone bioassays will be improved if laboratories conduct cone bioassays at consistent times of day, evening or night and report time of testing along with their results. When considering AIs that combat detoxification enzymes, conduct of tests during the dark phase will give more conservative and realistic estimations of efficacy since ITNs are designed to be used at night to sleep under.

The results are drawn from one facility; therefore, experiments from additional facilities with mosquitoes that have different levels of resistance, resistance profile and a greater range of mosquito species will contribute to the generalizability of the findings. Another limitation is that at the time of the experiment, the laboratory did not have metabolic resistant *Aedes* mosquitoes in the colony. This would have also contributed to the findings, since metabolically resistant *Ae. aegypti* have greater survival during the day, being the active phase for this diurnal species, so it would be expected that the day experiments have a more conservative estimate of mortality than those in the morning and evening. In addition, the experiments were conducted using only deltamethrin, and exploration of a broader range of insecticides is warranted.

## Conclusions

Time of day and monitoring delayed mortality should be considered when using WHO cone bioassays with metabolically resistant mosquitoes and testing those active ingredients that target them. When possible, cone bioassays may be conducted in the evening phase when nocturnal mosquitoes are likely to upregulate metabolic enzymes. Assessment of delayed mortality for insecticides should be consistent as different holding times will measure different levels of mortality. As such, mortality recorded from products may be either overestimated or underestimated depending on the time of day when the cone bioassays are performed and the length of holding time used. Accurately measuring insecticide efficacy is important to enable optimal products to be available for malaria control.

### Supplementary Information

**Additional file 1. **The origin and percentage 24-h mortality observed in the resistance profile of study test systems.

**Additional file 2. ** The influence of time of cone bioassay experiment on the 24-h mortality of laboratory-reared metabolic resistance, knockdown resistance and susceptible mosquitoes.

**Additional file 3. ** The influence of time of assessment of mortality of laboratory-reared metabolic resistance, knockdown resistance and susceptible mosquitoes on cone bioassay results.

**Additional file 4. ** Cone bioassay data on the time of exposure and assessment of mortality of laboratory-reared mosquitoes.

## Data Availability

Data are provided in Additional file 4.

## Abbreviations

AI: Active ingredient
CI: Confidence interval
DM: Deltamethrin
g: Gram
GLP: Good laboratory practice
HDPE: High-density polyethylene
IHI: Ifakara health institute
ITNs: Insecticide-treated nets
IRS: Indoor residual spraying
KDR: Knockdown resistance
kg: Kilogram
LSHTM: London School of Hygiene and Tropical Medicine
MR: Metabolic resistance
MR4: Malaria resource
M24: Mortality at 24 h
M72: Mortality at 72 h
OR: Odds ratio
PBO: Piperonyl butoxide
VCPTU: Vector Control Product Testing Unit
WHO: World Health Organization

## References

[1] Read AF, Lynch PA, Thomas MB (2009). How make evolution-proof insecticides for malaria control. PLOS Biol..

[2] Vanlerberghe V, Villegas E, Oviedo M, Baly A, Lenhart A, McCall PJ (2011). Evaluation of the effectiveness of insecticide treated materials for household level dengue vector control. PLoS Negl Trop Dis.

[3] Hougard JM, Corbel V, N'Guessan R, Darriet F, Chandre F, Akogbeto M (2003). Efficacy of mosquito nets treated with insecticide mixtures or mosaics against insecticide resistant *Anopheles gambiae* and *Culex quinquefasciatus* (Diptera: Culicidae) in Cote d'Ivoire. Bull Entomol Res.

[4] WHO (2022). World malaria report.

[5] FPemba D, Bandason E, Namangale J (2008). Comparison of deltamethrin as indoor residual spray or on insecticide treated nets for mosquito control in Lake Chilwa. Malawi Med J..

[6] Hawley WA, Phillips-Howard PA, Kuile FO, Terlouw DJ, Vulule JM, Ombok M (2003). Community-wide effects of permethrin-treated bed nets on child mortality and malaria morbidity in Western Kenya. Am Soc Trop Med Hyg..

[7] Levitz L, Janko M, Mwandagalirwa K, Thwai KL, Likwela JL, Tshefu AK (2018). Effect of individual and community-level bed net usage on malaria prevalence among under-fives in the Democratic Republic of Congo. Malar J.

[8] Nkya TE, Akhouayri I, Poupardin R, Batengana B, Mosha F, Magesa S (2014). Insecticide resistance mechanisms associated with different environments in the malaria vector *Anopheles gambiae*: a case study in Tanzania. Malar J.

[9] Abuelmaali SA, Elaagip AH, Basheer MA, Frah EA, Ahmed FT, Elhaj HF (2013). Impacts of agricultural practices on insecticide resistance in the malaria vector *Anopheles arabiensis* in Khartoum State, Sudan. PLoS ONE.

[10] Matowo NS, Tanner M, Munhenga G, Mapua SA, Finda M, Utzinger J (2020). Patterns of pesticide usage in agriculture in rural Tanzania call for integrating agricultural and public health practices in managing insecticide-resistance in malaria vectors. Malar J.

[11] Tepa A, Kengne-Ouafo JA, Djova VS, Tchouakui M, Mugenzi LMJ, Djouaka R (2022). Molecular drivers of multiple and elevated resistance to insecticides in a population of the malaria vector *Anopheles gambiae* in agriculture hotspot of west Cameroon. Genes (Basel)..

[12] van den Berg H, da Silva Bezerra HS, Al-Eryani S, Chanda E, Nagpal BN, Knox TB (2021). Recent trends in global insecticide use for disease vector control and potential implications for resistance management. Sci Rep.

[13] Nkya TE, Akhouayri I, Kisinza W, David J-P (2013). Impact of environment on mosquito response to pyrethroid insecticides: facts, evidences and prospects. Insect Biochem Mol Biol.

[14] WHO (2023). World malaria report.

[15] Martinez-Torres D, Chandre F, Williamson MS, Darriet F, Berge JB, Devonshire AL (1998). Molecular characterization of pyrethroid knockdown resistance (kdr) in the major malaria vector *Anopheles*
*gambiae* s.s. Insect Mol Biol..

[16] Vontas J, Katsavou E, Mavridis K (2020). Cytochrome P450-based metabolic insecticide resistance in *Anopheles* and *Aedes* mosquito vectors: Muddying the waters. Pestic Biochem Physiol.

[17] Balmert NJ, Rund SS, Ghazi JP, Zhou P, Duffield GE (2014). Time-of-day specific changes in metabolic detoxification and insecticide resistance in the malaria mosquito *Anopheles gambiae*. J Insect Physiol.

[18] Martin JL, Mosha FW, Lukole E, Rowland M, Todd J, Charlwood JD (2021). Personal protection with PBO-pyrethroid synergist-treated nets after 2 years of household use against pyrethroid-resistant *Anopheles* in Tanzania. Parasite Vectors.

[19] Protopopoff N, Mosha JF, Lukole E, Charlwood JD, Wright A, Mwalimu CD (2018). Effectiveness of a long-lasting piperonyl butoxide-treated insecticidal net and indoor residual spray interventions, separately and together, against malaria transmitted by pyrethroid-resistant mosquitoes: a cluster, randomised controlled, two-by-two factorial design trial. Lancet.

[20] WHO (2013). Guidelines for laboratory and field-testing of long-lasting insecticidal nets.

[21] WHO (2006). Guidelines for testing mosquito adulticides for indoor residual spraying and treatment of mosquito nets.

[22] WHO (2011). Guidelines for monitoring the durability of long-lasting insecticidal mosquito nets under operational conditions.

[23] WHO (2023). Guidelines for the prequalification assessment of insecticide treated nets.

[24] Mbwambo SG, Bubun N, Mbuba E, Moore J, Mbina K, Kamande D (2022). Comparison of cone bioassay estimates at two laboratories with different *Anopheles* mosquitoes for quality assurance of pyrethroid insecticide-treated nets. Malar J.

[25] Rund SS, O'Donnell AJ, Gentile JE, Reece SE (2016). Daily rhythms in mosquitoes and their consequences for malaria transmission. Insects..

[26] Hughes A, Matope A, Emery M, Steen K, Murray G, Ranson H (2022). A closer look at the WHO cone bioassay: video analysis of the hidden effects of a human host on mosquito behaviour and insecticide contact. Malar J.

[27] Koinari M, Bubun N, Amos B, Kiari K, Lahu D, Karl S (2022). WHO cone bioassay boards with or without holes: relevance for bioassay outcomes in long-lasting insecticidal net studies. Malar J.

[28] Owusu HF, Muller P (2016). How important is the angle of tilt in the WHO cone bioassay?. Malar J.

[29] Odufuwa OG, Moore SJ, Mboma ZM, Mbuba E, Muganga JB, Moore J (2022). Insecticide-treated eave nets and window screens for malaria control in Chalinze district, Tanzania: a study protocol for a household randomised control trial. Trials.

[30] Benedict MQ, Wilkins L, Howell P. MR4. Methods in *Anopheles* Research. bei Resources. 4th edition. 2014.

[31] Finda MF, Moshi IR, Monroe A, Limwagu AJ, Nyoni AP, Swai JK (2019). Linking human behaviours and malaria vector biting risk in South-eastern Tanzania. Plos One..

[32] StataCorp, (2019). Stata statistical software. Release 16.

[33] Šebesta O, Gelbič I, Peško J (2011). Daily and seasonal variation in the activity of potential vector mosquitoes. Open Life Sci.

[34] Meireles-Filho AC, Kyriacou CP (2013). Circadian rhythms in insect disease vectors. Mem Inst Oswaldo Cruz.

[35] Yang YY, Liu Y, Teng HJ, Sauman I, Sehnal F, Lee HJ (2010). Circadian control of permethrin-resistance in the mosquito *Aedes aegypti*. J Insect Physiol.

[36] Abdalla H, Matambo TS, Koekemoer LL, Mnzava AP, Hunt RH, Coetzee M (2008). Insecticide susceptibility and vector status of natural populations of *Anopheles arabiensis* from Sudan. Trans R Soc Trop Med Hyg.

[37] Liu N, Xu Q, Zhu F, Zhang L (2006). Pyrethroid resistance in mosquitoes. Insect Sci.

[38] Andreazza F, Oliveira EE, Martins GF (2021). Implications of sublethal insecticide exposure and the development of resistance on mosquito physiology, behavior, and pathogen transmission. Insects..

[39] Skovmand O, Dang DM, Tran TQ, Bossellman R, Moore SJ (2021). From the factory to the field: considerations of product characteristics for insecticide-treated net (ITN) bioefficacy testing. Malar J.

[40] Barreaux P, Koella JC, N’Guessan R, Thomas MB (2022). Use of novel lab assays to examine the effect of pyrethroid-treated bed nets on blood-feeding success and longevity of highly insecticide-resistant *Anopheles*
*gambiae* s.l. mosquitoes. Parasite Vectors..

[41] Huang P, Yan X, Yu B, He X, Lu L, Ren Y (2023). A comprehensive review of the current knowledge of chlorfenapyr: synthesis, mode of action, resistance, and environmental toxicology. Molecules..

[42] Wang C, Gourley SA, Liu R (2014). Delayed action insecticides and their role in mosquito and malaria control. J Math Biol.

[43] Glunt KD, Coetzee M, Huijben S, Koffi AA, Lynch PA, N'Guessan R (2018). Empirical and theoretical investigation into the potential impacts of insecticide resistance on the effectiveness of insecticide-treated bed nets. Evol Appl.

[44] Accrombessi M, Cook J, Dangbenon E, Yovogan B, Akpovi H, Sovi A (2023). Efficacy of pyriproxyfen-pyrethroid long-lasting insecticidal nets (LLINs) and chlorfenapyr-pyrethroid LLINs compared with pyrethroid-only LLINs for malaria control in Benin: a cluster-randomised, superiority trial. Lancet.

[45] Mosha JF, Kulkarni MA, Lukole E, Matowo NS, Pitt C, Messenger LA (2022). Effectiveness and cost-effectiveness against malaria of three types of dual-active-ingredient long-lasting insecticidal nets (LLINs) compared with pyrethroid-only LLINs in Tanzania: a four-arm, cluster-randomised trial. Lancet.

[46] Tambwe MM, Kibondo UA, Odufuwa OG, Moore J, Mpelepele A, Mashauri R (2023). Human landing catches provide a useful measure of protective efficacy for the evaluation of volatile pyrethroid spatial repellents. Parasite Vectors.

[47] WHO (2022). Standard operating procedure for determining the ability of PBO to restore susceptibility of adult mosquitoes to pyrethroid insecticides in WHO tube tests.

[48] Nikpour F, Vatandoost H, Hanafi-Bojd AA, Raeisi A, Ranjbar M, Enayati AA (2017). Evaluation of deltamethrin in combination of Piperonyl Butoxide (PBO) against pyrethroid resistant, malaria vector, *Anopheles stephensi* in IRS implementation: an experimental semi-filed trial in Iran. J Arthropod Borne Dis.

[49] Schmidt CA, Comeau G, Monaghan AJ, Williamson DJ, Ernst KC (2018). Effects of desiccation stress on adult female longevity in *Aedes*
*aegypti* and Ae. albopictus (Diptera: Culicidae) results of a systematic review and pooled survival analysis. Parasite Vectors..

