# Supplementary material for: Time of exposure and assessment influence the mortality induced by insecticides against metabolic resistant mosquitoes
Source: Parasit Vectors. 2024 Mar 2;17:103. doi: 10.1186/s13071-024-06190-z (PMC10908098; doi:10.1186/s13071-024-06190-z)
Supplement: Supplementary file 1 — Additional file 1. The origin and percentage 24-h mortality observed in the resistance profile of study test systems. [file 13071_2024_6190_MOESM1_ESM.docx]

| Test systems | Origin | Year of establishment in lHI lab | Time of test | Permethrin (0.75%) | Deltamethrin (0.05%) | Alpha-cypermethrin (0.05%) | Lambda-cyhalothorin (0.05%) | Pirimiphos methyl (0.25%) | PBO (4%) | Permethrin (0.75%) + PBO | Deltamethrin (0.05%) + PBO (4%) | Alpha-cypermethrin (0.05%) + PBO (4%) | Lambda-cyhalothorin (0.05%) + PBO (4%) |
| --- | --- | --- | --- | --- | --- | --- | --- | --- | --- | --- | --- | --- | --- |
| *An. arabiensis* (Kingani strain) | Ifakara, Tanzania | 2005 | November, 2020 | 13% | 25% | 16% | 26% | 100% | - | - | - | - | - |
|  |  |  | March 2022 | 11% | 22% | 17% | 21% | 98% | 5% | 99% | 100% | 100% | 100% |
| *An. funestus* (FUMOZ) | South Africa | 2021 | March 2022 | 60% | 78% | 72% | 66% | 100% | 6% | 100% | 100% | 100% | 100% |
| *An. gambiae*  (KDR, Kisumu strain) | Kisumu, Kenya | 2020 | March 2022 | 91% | 100% | 98% | 100% | 100% | 0% | - | - | - | - |
| *An. gambiae* (Susceptible, Ifakara strain) | Ifakara, Tanzania | 2021 | March 2022 | 100% | 100% | 100% | 100% | 100% | 0% | - | - | - | - |
| *Culex quinquefasciatus* (Bagamoyo strain) | Bagamoyo, Tanzania | 2019 | November, 2020 | 14% | 20% | 13% | 21% | 83% | - | - | - | - | - |
| *Aedes aegypti* (Bagamoyo strain) | Bagamoyo, Tanzania | 2015 | November, 2020 | 100% | 100% | 100% | 100% | 74% | - | - | - | - | - |

- Denotes that tests were not conducted at that time.
