# Supplementary material for: Time of exposure and assessment influence the mortality induced by insecticides against metabolic resistant mosquitoes
Source: Parasit Vectors. 2024 Mar 2;17:103. doi: 10.1186/s13071-024-06190-z (PMC10908098; doi:10.1186/s13071-024-06190-z)
Supplement: Supplementary file 2 — Additional file 2. The influence of time of cone bioassay experiment on the 24-h mortality of laboratory-reared metabolic resistance, knockdown resistance and susceptible mosquitoes. [file 13071_2024_6190_MOESM2_ESM.docx]

| Mosquitoes | Insecticides/synergy | Time | Total exposed | Total dead | %M24  (95% C I) | ^1a^OR  (95% CI) | P-value |
| --- | --- | --- | --- | --- | --- | --- | --- |
| *An. arabiensis* (Kingani strain, metabolic resistant) | Deltamethrin  (ITENs & ITWS) | Day | 120 | 115 | 95.8 (92.5 - 99.2) | 1.00 |  |
|  |  | Evening | 120 | 101 | 84.2 (77.5 - 90.8) | 0.23 (0.08 – 0.64) | 0.005 |
|  | Deltamethrin & PBO  (ITENs & ITWS) | Day | 120 | 102 | 85.0 (78.6 - 91.4) | 1.00 |  |
|  |  | Evening | 120 | 85 | 70.8 (63.8 - 77.9) | 0.43 (0.23 – 0.81) | 0.009 |
|  | Deltamethrin (ITNs) | Afternoon | 120 | 120 | 100 | 1.00 |  |
|  |  | Night | 120 | 120 | 100 | 1.00 | - |
|  | Pre-exposed to PBO + Deltamethrin (ITNs) | Afternoon | 120 | 120 | 100 | 1.00 |  |
|  |  | Night | 120 | 120 | 100 | 1.00 | - |
| *Cx. Quinquefasciatus*  (Bagamoyo strain, metabolic resistant) | Deltamethrin  (ITENs & ITWS) | Day | 180 | 26 | 14.4 (8.7 - 20.2) | 1.00 |  |
|  |  | Evening | 180 | 2 | 1.1 (0 - 2.6) | 0.07 (0.02 – 0.28) | <0.0001 |
|  | Deltamethrin & PBO  (ITENs & ITWS) | Day | 180 | 29 | 16.1 (11.0 - 21.3) | 1.00 |  |
|  |  | Evening | 180 | 5 | 2.8 (0.5 - 5.1) | 0.15 (0.06 – 0.39) | <0.0001 |
|  | Deltamethrin (ITNs) | Afternoon | 120 | 13 | 10.8 (5.1 - 16.6) | 1.00 |  |
|  |  | Night | 120 | 7 | 5.8 (1.4 - 10.2) | 0.51 (0.20 - 1.33) | 0.167 |
|  | Pre-exposed to PBO + Deltamethrin (ITNs) | Afternoon | 120 | 36 | 30.0 (20.3 - 39.7) | 1.00 |  |
|  |  | Night | 120 | 33 | 27.5 (19.0 - 36.0) | 0.89 (0.51 - 1.55) | 0.669 |
| *An. funestus* (FUMOZ strain, metabolic resistant) | Deltamethrin (ITNs) | Afternoon | 120 | 116 | 96.7 (93.6 - 99.7) | 1.00 |  |
|  |  | Night | 120 | 107 | 89.2 (83.4 - 94.9) | 0.28 (0.09 – 0.90) | 0.032 |
|  | Pre-exposed to PBO + Deltamethrin (ITNs) | Afternoon | 120 | 120 | 100 | 1.00 |  |
|  |  | Night | 120 | 120 | 100 | 1.00 | - |
| *An. gambiae*  (Kisumu strain, *KDR*) | Deltamethrin (ITNs) | Afternoon | 120 | 114 | 95.0 (90.1 - 99.9) | 1.00 |  |
|  |  | Night | 120 | 119 | 99.2 (97.5 - 100) | 6.26 (0.74 – 52.84) | 0.092 |
|  | Pre-exposed to PBO + Deltamethrin (ITNs) | Afternoon | 120 | 120 | 100 | 1.00 |  |
|  |  | Night | 120 | 120 | 100 | 1.00 | - |
| *An. gambiae*  (Ifakara strain, Susceptible) | Deltamethrin (ITNs) | Afternoon | 120 | 120 | 100 | 1.00 |  |
|  |  | Night | 120 | 120 | 100 | 1.00 | - |
|  | Pre-exposed to PBO + Deltamethrin (ITNs) | Afternoon | 120 | 120 | 100 | 1.00 |  |
|  |  | Night | 120 | 120 | 100 | 1.00 | - |
| *Ae. aegypti*  (Bagamoyo strain, susceptible) | Deltamethrin  (ITENs & ITWS) | Day | 120 | 120 | 100 | 1.00 |  |
|  |  | Evening | 120 | 120 | 100 | 1.00 | - |
|  | Deltamethrin & PBO  (ITENs & ITWS) | Day | 120 | 94 | 78.3 (70.5 - 86.1) | 1.00 |  |
|  |  | Evening | 120 | 86 | 71.7 (61.9 - 81.4) | 0.70 (0.39 - 1.26) | 0.234 |
|  | Deltamethrin (ITNs) | Afternoon | 120 | 120 | 100 | 1.00 |  |
|  |  | Night | 120 | 120 | 100 | 1.00 | - |
|  | Pre-exposed to PBO + Deltamethrin (ITNs) | Afternoon | 120 | 120 | 100 | 1.00 |  |
|  |  | Night | 120 | 120 | 100 | 1.00 | - |

***1^a^Estimate for the effect of hour of cone bioassay experiment on mortality, using logistic regression. Day is the reference for evening and afternoon is the reference for night. Overall mortality in the negative control was 0% at 24 hours.***
