# Supplementary material for: Time of exposure and assessment influence the mortality induced by insecticides against metabolic resistant mosquitoes
Source: Parasit Vectors. 2024 Mar 2;17:103. doi: 10.1186/s13071-024-06190-z (PMC10908098; doi:10.1186/s13071-024-06190-z)
Supplement: Supplementary file 3 — Additional file 3. The influence of time of assessment of mortality of laboratory-reared metabolic resistance, knockdown resistance and susceptible mosquitoes on cone bioassay results. [file 13071_2024_6190_MOESM3_ESM.docx]

| Mosquitoes | Insecticides/synergy | | Time | Total exposed | Total dead | % Mortality  (95% CI) | ^1b^OR  (95% CI) | | P-value |
| --- | --- | --- | --- | --- | --- | --- | --- | --- | --- |
| *An. arabiensis* (Kingani strain, metabolic resistant) | Deltamethrin  (ITENs & ITWS) | | 24 hr. | 240 | 216 | 90.0 (86.0 - 94.0) | 1.00 | |  |
|  |  |  | 72 hr. | 240 | 222 | 92.5 (88.9 – 96.1) | 1.39 (0.72 – 2.66) | | 0.325 |
|  | Deltamethrin & PBO  (ITENs & ITWS) | | 24 hr. | 240 | 187 | 77.9 (72.8 –83.0) | 1.00 | |  |
|  |  |  | 72 hr. | 240 | 204 | 85.0 (79.8 – 90.2) | 1.63 (1.01 – 2.62) | | 0.044 |
|  | Deltamethrin (ITNs) | | 24 hr. | 240 | 240 | 100 | 1.00 | |  |
|  |  |  | 72 hr. | 240 | 240 | 100 | 1.00 | | - |
|  | Pre-exposed to PBO + Deltamethrin (ITNs) | | 24 hr. | 240 | 240 | 100 | 1.00 | |  |
|  |  |  | 72 hr. | 240 | 240 | 100 | 1.00 | | - |
| *Cx. Quinquefasciatus*  (Bagamoyo strain, metabolic resistant) | Deltamethrin  (ITENs & ITWS) | | 24 hr. | 360 | 28 | 7.8 (4.4 – 11.1) | 1.00 | |  |
|  |  |  | 72 hr. | 360 | 60 | 16.7 (12.9 – 20.4) | 2.54 (1.55 – 4.15) | | <0.0001 |
|  | Deltamethrin & PBO  (ITENs & ITWS) | | 24 hr. | 360 | 34 | 9.4 (6.2 – 12.6) | 1.00 | |  |
|  |  |  | 72 hr. | 360 | 93 | 25.8 (22.4 – 29.3) | 3.45 (2.24 – 5.30) | | <0.0001 |
|  | Deltamethrin (ITNs) | | 24 hr. | 240 | 20 | 8.3 (4.7 - 12.0) | 1.00 | |  |
|  |  |  | 72 hr. | 240 | 22 | 9.2 (5.0 - 13.4) | 1.11 (0.59 – 2.10) | | 0.745 |
|  | Pre-exposed to PBO + Deltamethrin (ITNs) | | 24 hr. | 240 | 69 | 28.8 (22.4 - 35.1) | 1.00 | |  |
|  |  |  | 72 hr. | 240 | 92 | 38.3 (31.1 - 45.6) | 1.54 (1.05 – 2.26) | | 0.026 |
| *An. funestus* (FUMOZ strain, metabolic resistant) | Deltamethrin (ITNs) | | 24 hr. | 240 | 223 | 92.9 (89.5 - 96.3) | 1.00 | |  |
|  |  |  | 72 hr. | 240 | 234 | 97.5 (95.3 - 99.7) | 2.99 (1.15 – 7.73) | | 0.024 |
|  | Pre-exposed to PBO + Deltamethrin (ITNs) | | 24 hr. | 240 | 240 | 100 | 1.00 | |  |
|  |  |  | 72 hr. | 240 | 240 | 100 | 1.00 | | - |
| *An. gambiae*  (Kisumu strain, *KDR*) | Deltamethrin (ITNs) | | 24 hr. | 240 | 233 | 97.1 (94.5 - 99.7) | 1.00 | |  |
|  |  |  | 72 hr. | 240 | 237 | 98.8 (96.9 - 100) | 2.40 (0.61 – 9.47) | | 0.211 |
|  | Pre-exposed to PBO + Deltamethrin (ITNs) | | 24 hr. | 240 | 240 | 100 | 1.00 | |  |
|  |  |  | 72 hr. | 240 | 240 | 100 | 1.00 | | - |
| *An. gambiae*  (Ifakara strain, Susceptible) | | Deltamethrin (ITNs) | 24 hr. | 240 | 240 | 100 | 1.00 |  | |
|  |  |  | 72 hr. | 240 | 240 | 100 | 1.00 | - | |
|  |  | Pre-exposed to PBO + Deltamethrin (ITNs) | 24 hr. | 240 | 240 | 100 | 1.00 |  | |
|  |  |  | 72 hr. | 240 | 240 | 100 | 1.00 | - | |
| *Ae. aegypti*  (Bagamoyo strain, susceptible) | | Deltamethrin  (ITENs & ITWS) | 24 hr. | 240 | 240 | 100 | 1.00 |  | |
|  |  |  | 72 hr. | 240 | 240 | 100 | 1.00 | - | |
|  |  | Deltamethrin & PBO  (ITENs & ITWS) | 24 hr. | 240 | 180 | 75.0 (68.8 – 81.2) | 1.00 |  | |
|  |  |  | 72 hr. | 240 | 169 | 70.4 (64.2 – 76.6) | 0.79 (0.53 – 1.19) | 0.260 | |
|  |  | Deltamethrin (ITNs) | 24 hr. | 240 | 240 | 100 | 1.00 |  | |
|  |  |  | 72 hr. | 240 | 240 | 100 | 1.00 | - | |
|  |  | Pre-exposed to PBO + Deltamethrin (ITNs) | 24 hr. | 240 | 240 | 100 | 1.00 |  | |
|  |  |  | 72 hr. | 240 | 240 | 100 | 1.00 | - | |

***1^b^Estimate for the effect of hour of assessment on mortality, using logistic regression with time of cone bioassay set as a fixed effect. 24 hour is the reference. Overall mortality in the negative control was 0% and 1% at 72 hours for An. funestus only.***
